# Response of Unvaccinated US Adults to Official Information About the Pause in Use of the Johnson & Johnson–Janssen COVID-19 Vaccine: Cross-Sectional Survey Study

**DOI:** 10.2196/41559

**Published:** 2024-04-01

**Authors:** Vishala Mishra, Joseph P Dexter

**Affiliations:** 1 Department of Biostatistics and Bioinformatics Duke University School of Medicine Durham, NC United States; 2 Data Science Initiative Harvard University Allston, MA United States; 3 Department of Human Evolutionary Biology Harvard University Cambridge, MA United States; 4 Institute of Collaborative Innovation University of Macau Taipa Macao

**Keywords:** Centers for Disease Control and Prevention, CDC, COVID-19, health communication, health information, health literacy, public health, risk perception, SARS-CoV-2, vaccine hesitancy, web-based surveys

## Abstract

Using a rapid response web-based survey, we identified gaps in public understanding of the Centers for Disease Control and Prevention’s messaging about the pause in use of the Johnson & Johnson–Janssen COVID-19 vaccine and estimated changes in vaccine hesitancy using counterfactual questions.

## Introduction

On April 13, 2021, the Centers for Disease Control and Prevention (CDC) and Food and Drug Administration recommended a pause in use of the Johnson & Johnson (J&J)–Janssen COVID-19 vaccine due to 6 reports of cerebral venous sinus thrombosis in recently vaccinated individuals [1]. The announcement of the pause required development of a coordinated communication strategy under extreme time pressure and careful messaging by stakeholders to mitigate reduced public confidence in COVID-19 vaccines [2]. Moreover, official communication efforts had to consider the potential influence of already widespread misinformation about the vaccines on attitudes toward the pause [3,4]. In this survey study, we evaluated understanding and impressions of the CDC’s public web-based information about the J&J-Janssen pause among unvaccinated US adults.

## Methods

### Web-Based Survey About J&J-Janssen Pause

We administered the web-based survey to two cohorts of US adults recruited through Prolific between April 19-21, 2021 (cohort A), and April 21-23, 2021 (cohort B). Both cohorts were assembled using convenience sampling of unvaccinated adults. To obtain information about a population that especially needed targeted vaccine communication, the first cohort was restricted to individuals expressing neutral or negative sentiments about COVID-19 vaccines. The survey design and recruitment strategy are described in Multimedia Appendix 1; the survey questions are provided in Multimedia Appendices 2 and 3.

### Ethical Considerations

The study was approved by Harvard University’s Committee on the Use of Human Subjects (IRB20-2089), and participants agreed to a consent statement on the first page of the survey. Participants were paid US $2 for taking the survey. All study data were collected anonymously.

## Results

A total of 271 and 286 participants were included in cohorts A and B, respectively (demographic characteristics listed in Table 1). Across participants, the median number of correct responses to the comprehension questions was 6 in both cohort A (IQR 1.5; range 0-7) and cohort B (IQR 1.0; range 1-7). The total number of correct responses was negatively associated with intention not to seek vaccination in both cohort A (odds ratio 0.61, 95% CI 0.45-0.82; *P*=.001) and cohort B (odds ratio 0.48, 95% CI 0.31-0.74; *P*=.001; Multimedia Appendix 4). Although a majority of participants rated the passages as “clear and easy to read” (cohort A: n=229, 84.5%; cohort B: n=243, 85%), fewer indicated that they would be likely to share them on social media (cohort A: n=53, 19.6%; cohort B: n=75, 26.3%).

**Figure 1:**
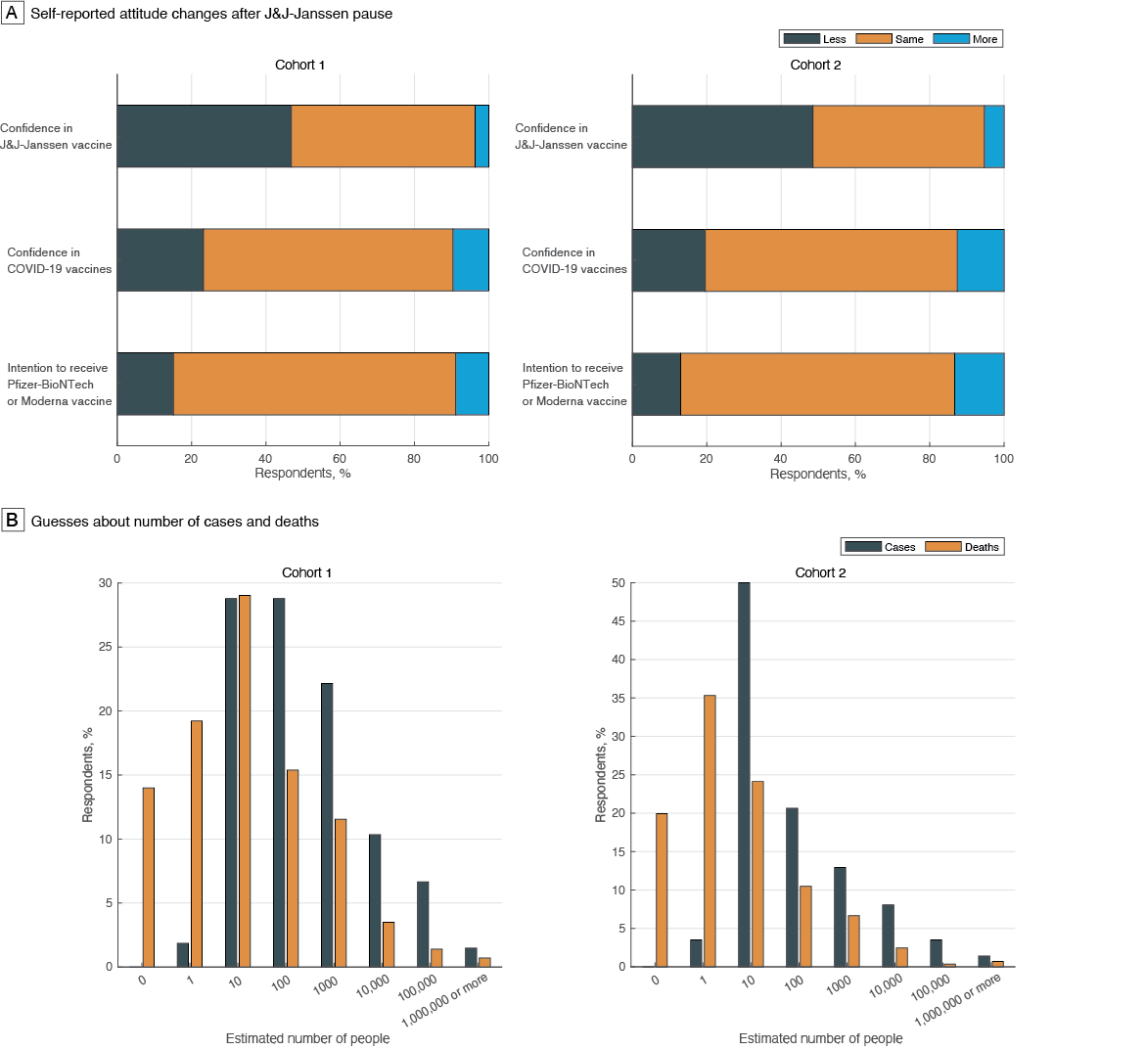
Self-reported attitude changes and estimated cases and deaths. J&J: Johnson & Johnson.

The web page mentioned “a small number of reports” of cerebral venous sinus thrombosis in individuals who received the J&J-Janssen vaccine. When asked to guess a specific number, 188 (69.4%) and 133 (46.5%) respondents in cohorts A and B, respectively, estimated 100 or more cases, at least an order of magnitude higher than the actual value; 176 (64.9%) and 128 (44.8%) respondents in cohorts A and B, respectively, estimated 10 or more deaths after vaccination (Figure 1).

Responding to a counterfactual question, 127 (46.9%) and 139 (48.6%) participants in cohorts A and B, respectively, indicated that the pause reduced their confidence in the J&J-Janssen vaccine’s safety (Figure 1). Most participants reported no change in their confidence in COVID-19 vaccines’ safety in general (cohort A: n=182, 67.2%; cohort B: n=194, 67.8%) or intention to receive the Pfizer-BioNTech or Moderna vaccine (cohort A: n=206, 76%; cohort B: n=211, 73.8%).

**Table 1:** Characteristics and responses of participants who completed the web-based surveys about the Johnson & Johnson (J&J)–Janssen vaccine pause.

Characteristics			Cohort A (n=271), n (%)		Cohort B (n=286), n (%)
**Age (years)**					
	18-29	119 (43.9)		145 (50.7)	
	30-49	116 (42.8)		111 (38.9)	
	≥50	36 (13.3)		30 (10.5)	
**Gender**					
	Female	144 (53.1)		136 (47.6)	
	Male	124 (45.8)		144 (50.3)	
	Nonbinary, transgender, or other	3 (1.1)		6 (2.1)	
Ethnicity: Hispanic or Latinx			54 (20.0)		39 (13.6)
**Race^a^**					
	Asian	29 (10.7)		96 (33.6)	
	Black or African American	70 (25.8)		37 (12.9)	
	White	160 (59.0)		139 (48.6)	
	Other^b^	27 (10.0)		28 (9.8)	
**Educational attainment**					
	Less than high school diploma	3 (1.1)		6 (2.1)	
	High school diploma or equivalent	54 (19.9)		46 (16.1)	
	Some college or associate’s degree	102 (37.6)		106 (37.1)	
	Bachelor’s degree	81 (29.9)		102 (35.7)	
	Graduate or professional degree	31 (11.4)		26 (9.1)	
**Political partisanship**					
	Democratic (including leaners)	81 (29.9)		150 (52.4)	
	Republican (including leaners)	91 (33.6)		42 (14.7)	
	Independent or other	99 (36.5)		94 (32.9)	
**Geography**					
	Rural area	41 (15.1)		35 (12.2)	
	Suburban or urban area	230 (84.9)		251 (87.8)	
**Intention to receive vaccine**					
	Definitely will not	71 (26.2)		29 (10.1)	
	Probably will not	68 (25.1)		17 (5.9)	
	Undecided	54 (19.9)		29 (10.1)	
	Definitely or probably will	78 (28.8)		211 (73.8)	
**Comprehension questions^c^**					
	Reason for pause	259 (95.6)		280 (97.9)	
	Causal relationship between vaccine and side effect	196 (72.3)		215 (75.2)	
	Affected population	234 (86.3)		248 (86.7)	
	Safety of mRNA vaccines	214 (79.0)		250 (87.4)	
	Revaccination of J&J-Janssen recipients	164 (60.5)		189 (66.1)	
	Symptom monitoring for J&J-Janssen recipients	180 (66.4)		121 (42.3)	
	Rescheduling of canceled appointments	192 (70.8)		229 (80.1)	
**Comprehension score^d^**					
	7	62 (22.9)		43 (15.0)	
	6	80 (29.5)		119 (41.6)	
	5	61 (22.5)		59 (20.6)	
	4	33 (12.2)		38 (13.3)	
	3	24 (8.9)		18 (6.3)	
	0-2	11 (4.1)		9 (3.1)	
**Self-reported impressions of passage^e^**					
	Accurate and should be trusted	119 (43.9)		198 (69.2)	
	High-quality evidence	110 (40.6)		180 (62.9)	
	Clear and easy to read	229 (84.5)		243 (85.0)	
	Understandable	229 (84.5)		251 (87.8)	
	Requiring great effort to understand	58 (21.4)		69 (24.1)	
	Other people would want to read	173 (63.8)		172 (60.1)	
	Would share on social media	53 (19.6)		75 (26.2)	

^a^Participants could select more than one option.

^b^Includes participants who selected “American Indian or Alaska Native,” “Native Hawaiian or Other Pacific Islander,” or “Another option not listed here.”

^c^Number of participants who gave the correct answer to each question.

^d^Number of participants who gave the indicated number of correct answers across all questions.

^e^Number of participants who answered “Strongly agree” or “Agree” about each description on a 6-point Likert scale.

## Discussion

In our web-based survey about the CDC’s messaging around the J&J-Janssen vaccine pause, many respondents overestimated the number of case reports that prompted the pause, often by several orders of magnitude. Since verbal descriptors are elastic concepts that can be misinterpreted, grounding them with numbers can reduce variability in risk perception and promote informed decision-making [5].

Respondents also expressed reduced confidence in the safety of the J&J-Janssen vaccine, highlighting the potential danger of conveying piecemeal information about risk during a pandemic response [3]. Encouragingly, the reduced confidence did not extend to mRNA COVID-19 vaccines, consistent with previous findings that overall vaccine hesitancy remained stable following the pause [6]. These results were obtained using the counterfactual format, which is less susceptible to overestimating shifts in beliefs than the change format (Multimedia Appendix 1). The negative association between understanding of the passage and self-reported vaccine hesitancy suggests that more targeted messaging may have been useful to promote vaccine confidence [7,8].

Consistent with uncertainty management theory [9], individuals likely viewed the pause in different ways, leading to a spectrum of emotional responses and changes in behavior. Despite being a safety precaution, the pause introduced new uncertainties requiring effective management through clear and consistent messaging, highlighting the balance that must be maintained between fostering trust and preventing unnecessary alarm [10]. Given the limitations of the deficit model of scientific communication [11], just providing technically correct information is insufficient for effective communication during public health crises. Instead, attention should be given to the accessibility of information across diverse socioeconomic groups, in line with the knowledge gap hypothesis [12], and to countering misinformation by providing easy-to-use official guidance [6,7].

The study is limited by the convenience sampling strategy; the participants recruited were not representative of the US population as a whole, and the findings should not be generalized to other contexts. Since the study was conducted on the web, individuals with lower internet and health literacy may have been excluded.

## Appendix

Multimedia Appendix 1 Additional information about survey methodology.

Multimedia Appendix 2 Survey administered to cohort A.

Multimedia Appendix 3 Survey administered to cohort B.

Multimedia Appendix 4 Supplemental tables about survey questions and ordinal logistic regression analysis.

## Abbreviations

CDC: Centers for Disease Control and Prevention
J&J: Johnson & Johnson

## Data Availability

The data sets generated during this study are available from the corresponding author upon reasonable request.
