# Supplementary material for: Response of Unvaccinated US Adults to Official Information About the Pause in Use of the Johnson & Johnson–Janssen COVID-19 Vaccine: Cross-Sectional Survey Study
Source: J Med Internet Res. 2024 Apr 1;26:e41559. doi: 10.2196/41559 (PMC11019423; doi:10.2196/41559)
Supplement: Multimedia Appendix 1 [file jmir_v26i1e41559_app1.pdf]

Multimedia Appendix 1: Additional information about survey methodology

Survey Passages

The survey passages were taken from the CDC's primary web page for communicating information about the pause to the general public [1,2]. To create the text included in the survey, we removed links and references to other web pages and all formatting other than section headers, bulleted lists, and paragraph breaks. No other changes were made to the passages.

Participants in cohort A were presented with the version of the passage released on April 16, 2021. It is 948 words long and scores above an eighth-grade reading level by both the Simple Measure of Gobbledygook (SMOG) and Flesch-Kincaid Grade Level (FKGL) metrics (12.9 and 11.4, respectively). On April 20, 2021, the CDC made several changes to the passage but did not modify the core content. Changes included reducing the length to 795 words and altering the language and section headings. Participants in cohort B were given the revised passage, which scores 12.7 by SMOG and 10.7 by FKGL.

The passages and survey questions are provided in Multimedia Appendix 2 and Multimedia Appendix 3.

Assessment of COVID-19 Vaccine Intention

The survey included two questions about COVID-19 vaccine intention adapted from Persad *et al*. 2021 [3], which were presented before the passage. The first question asked "How likely are you to get vaccinated for COVID-19 when a vaccine is available for you?" and included 6 answer choices ("I definitely will NOT get vaccinated."; "I probably will NOT get vaccinated."; "I probably will get vaccinated."; "I definitely will get vaccinated."; "I am completely undecided about whether I will get vaccinated."; "I have already been vaccinated.") The second question asked "Thinking about this in a different way, which of the following statements comes closest to what you are most likely to do when a COVID-19 vaccine is available for you?" and included 4 answer choices ("I will get vaccinated as soon as possible."; "I will wait to see what happens with other people before deciding whether to get vaccinated myself."; "I will not get vaccinated, regardless of what happens to other people who get the vaccine."; "I have already been vaccinated.")

Comprehension Questions

We developed 7 multiple-choice questions to assess how participants understood the major recommendations of the passage. The full set of questions and answer choices, along with participant responses, are provided in Table 1. The first 3 questions involve recall of information stated directly in the passage; the remaining 4 questions involve application of concepts to hypothetical scenarios.

Impression Questions

Participants were asked to rate their impressions of 7 attributes of the passage using a 6-point Likert scale (options "Strongly disagree"; "Disagree"; "Slightly disagree"; "Slightly agree"; "Agree"; "Strongly agree"). The questions were adapted from a previous study of COVID-19 health literacy by Kerr *et al*. 2021 [4].

Counterfactual Questions

We used the nonrandomized counterfactual format described by Graham and Coppock 2021 to assess participants' beliefs about the effect of the J&J-Janssen pause on their confidence in the safety of COVID-19 vaccines and the likelihood that they would receive the Pfizer-BioNTech or Moderna vaccine [1,2]. The counterfactual format is an alternative to the standard change format, in which participants are asked directly about changes in their beliefs. Although widely used, the change format can yield biased results because respondents often report their current attitudes instead of the change (response substitution). In the counterfactual format, participants are asked about their beliefs assuming various hypothetical scenarios; differences between their hypothetical and actual attitudes provide insight into perceived causal effects [1,2]. In our survey, participants first answered three questions about their current beliefs after reading the passage. They were then instructed to answer the same three questions as if they had been asked before the pause was announced. For Figure 1, we tabulated the number of participants who expressed more, less, or the same confidence in response to the counterfactual questions than the baseline questions.

Estimation Questions

Participants were also asked to estimate the number of cases of and deaths due to CVST that motivated the pause, which was not stated in the passage, as well as how long they anticipated the pause to last. These questions were intended to elicit information about the public's risk perception during a period of substantial uncertainty [5].

Demographic Information

Participants self-reported demographic information about age, gender, ethnicity, race, educational attainment, political partisanship and ideology, and place of residence.

Prescreening and Sampling Strategy

We administered the surveys through Prolific (https://www.prolific.co/), an online survey vendor with a large number of active users in the United States. Both cohorts were assembled using a convenience sampling strategy in which participants enrolled on a first-come, first-served basis. Prescreening questions written by Prolific and previously administered to their users were employed to restrict enrollment to the target populations.

The following prescreening criteria were applied to both cohorts:

Question: "In which country do you currently reside?"

Accepted answer: "United States"

Question: "Have you received a coronavirus (COVID-19) vaccination?"

Accepted answer: “No”

One additional criterion regarding vaccine hesitancy was applied only to cohort A:

Question: “Please describe your attitudes towards the COVID-19 (Coronavirus) vaccines:”

Accepted answers: “Against (I feel negatively about the vaccines)”; “Neutral (I don’t have strong opinions either way)”

In addition, participants in related pilot studies run by the investigators between February 1, 2021 and April 15, 2021 were excluded during prescreening, and all participants in cohort A were ineligible to join cohort B.

To increase the racial and ethnic diversity of the convenience samples, we limited the number of participants who could enroll in each cohort based on their answers to the following prescreening question:[4]

“Please indicate your ethnicity (i.e. peoples’ ethnicity describes their feeling of belonging and attachment to a distinct group of a larger population that shares their ancestry, colour, language or religion)?”

For each cohort, we capped enrollment at a maximum of 100 people for each of the following 5 groups:

Group 1: Responded “White/Caucasian”
Group 2: Responded “Black/African American”
Group 3: Responded “East Asian”, “South Asian”, or “South East Asian”
Group 4: Responded “African”, “Caribbean”, “Middle Eastern”, “Mixed”, “Native American or Alaskan Native”, “Other (please feel free to us know your ethnicity via email)”, “White/Sephardic Jew”, “Black/British”, “White Mexican”, or “Romani/Traveller”
Group 5: Responded “Latino/Hispanic”

Exclusion Criteria

303 participants enrolled in cohort A, and 286 participants enrolled in cohort B, which was close to the maximum number of eligible individuals we could recruit on Prolific within two days of survey launch. After data collection, we excluded participants who met any of the following criteria:

1) Did not complete the whole survey.

2) Answered “Not at all carefully” to the question “How carefully did you complete this survey? Please answer honestly. Your payment does NOT depend on your response to this question.”

3) Gave incorrect responses to both attention check questions, “Please answer ‘Slightly unlikely’ to this question.” and “What color is the sky? Please answer this question incorrectly, on purpose, by choosing ‘Red’ instead of ‘Blue.’ ”

4) Answered “Yes, one dose” or “Yes, two doses” to the question “Have you gotten a COVID-19 vaccine?”

In addition, participants were excluded from cohort A if they reported a strong intention to receive a COVID-19 vaccine, in contrast to their answers to the Prolific prescreening questions. For these purposes, strong intention was defined as answering “I definitely will get vaccinated” to the question “How likely are you to get vaccinated for COVID-19 when a vaccine is available for you?” and answering “I will get vaccinated as soon as possible” to the question “Thinking about this in a different way, which of the following statements comes closest to what you are most likely to do when a COVID-19 vaccine is available for you?”

Out of cohort A, 32 participants were excluded, all for reasons relating to vaccination history or intentions. No participants were excluded from cohort B. Participants who reported using Google searches or other outside help to answer the survey questions (9 [3.3%] in cohort A and 10 [3.5%] in cohort B) were not excluded, as overall use of outside resources was limited, and these participants all passed other attention checks.

Statistical Analysis

Associations between participant characteristics and performance on the comprehension questions were assessed using ordinal logistic regression. The dependent variable was the total number of correct responses to the 7 comprehension questions. Statistical analyses were performed using the Python package statsmodels (version 0.13.0.dev0). Statistical significance was defined as $P < .05$.

References

1. Graham MH, Coppock A. Asking about attitude change. Public Opin Q. 2021;85(1):28-53.
2. Graham M, Coppock A. How to use the counterfactual polling format to ask about attitude change. 2021. URL: https://alexandercoppock.com/subpages/counterfactual_format.html [accessed 06-06-2021]
3. Persad G, Emanuel EJ, Sangenito S, Glickman A, Phillips S, Largent EA. Public perspectives on COVID-19 vaccine prioritization. JAMA Netw Open. 2021;4(4):e217943.
4. Kerr JR, Freeman ALJ, Marteau TM, van der Linden S. Effect of information about COVID-19 vaccine effectiveness and side effects on behavioural intentions: Two online experiments. Vaccines. 2021:9(4):379.
5. Lichtenstein S, Slovic P, Fischhoff B, Layman, M, Combs B. Judged frequency of lethal events. J Exp Psychol: Learn Mem Cogn. 1978;4(6):551-578.
