# Supplementary material for: Response of Unvaccinated US Adults to Official Information About the Pause in Use of the Johnson & Johnson–Janssen COVID-19 Vaccine: Cross-Sectional Survey Study
Source: J Med Internet Res. 2024 Apr 1;26:e41559. doi: 10.2196/41559 (PMC11019423; doi:10.2196/41559)
Supplement: Multimedia Appendix 3 [file jmir_v26i1e41559_app3.pdf]

Multimedia Appendix 3: Survey administered to cohort B

Please answer the following questions about your experiences with the COVID-19 pandemic and COVID-19 vaccines.

Around how often do you look for information about COVID-19 on the internet?
Never
Once a month
2-3 times per month
Once a week
Multiple times per week
Once a day
Multiple times per day

Do you ever share information about COVID-19 with your social media network?

No
Yes
I do not have an active account on a social media platform.

Which cable news network do you watch most often?

CNN
Fox News
International news channels (such as BBC, Al Jazeera, or Euronews)
MSNBC
I do not watch cable news.

As far as you know, do you have COVID-19 now, or have you had COVID-19 in the past?

No
Yes
I don't know.

As far as you know, has anyone in your immediate social circle, such as close friends or family members, had COVID-19?

No
Yes
I don't know.

Are you currently eligible to get a COVID-19 vaccine where you live?

No
Yes
I don't know.

Have you gotten a COVID-19 vaccine?

No
Yes, one dose
Yes, two doses

How likely are you to get vaccinated for COVID-19 when a vaccine is available for you?

I definitely will NOT get vaccinated.
I probably will NOT get vaccinated.
I probably will get vaccinated.
I definitely will get vaccinated.
I am completely undecided about whether I will get vaccinated.
I have already been vaccinated.

Thinking about this in a different way, which of the following statements comes closest to what you are most likely to do when a COVID-19 vaccine is available for you?

I will get vaccinated as soon as possible.
I will wait to see what happens with other people before deciding whether to get vaccinated myself.
I will not get vaccinated, regardless of what happens to other people who get the vaccine.
I have already been vaccinated.

Have you scheduled a COVID-19 vaccine appointment?

Yes, I already got a COVID-19 vaccine.
Yes, I have an upcoming COVID-19 vaccine appointment.
Yes, but my COVID-19 vaccine appointment was cancelled.
No, I don't know how to schedule a COVID-19 vaccine appointment.

No, I am not yet eligible to get a COVID-19 vaccine.
No, I don't plan to schedule a COVID-19 vaccine appointment.

Below are some reasons people may give for why they will NOT get a COVID-19 vaccine. For each one, please indicate whether it definitely applies to you, somewhat applies to you, or does not apply to you as a reason for not getting a COVID-19 vaccine. There is NO right or wrong answer. We are only interested in your personal opinion.

	Definitely applies to me	Somewhat applies to me	Does not apply to me
I have a health condition that would make it risky for me to get a vaccine.	o	o	o
I have a right not to get vaccinated and choose to exercise that right.	o	o	o
I do not trust vaccines in general.	o	o	o
I do not think the vaccine will be effective in protecting me from COVID-19.	o	o	o
I do not think the vaccine will be safe because it could have harmful side effects.	o	o	o
I am concerned approval of the vaccine has been rushed, without	o	o	o

adequate testing for safety and effectiveness.			
I do not think it is necessary to get vaccinated because COVID-19 is not as big of a problem as it is being made out to be.	o	o	o
I do not think it is necessary to get vaccinated because I take precautions to protect myself, such as wearing a mask, maintaining physical distance, and washing hands often.	o	o	o
I do not think I will get very sick if I get COVID-19.	o	o	o
I am not able to get a COVID-19 vaccine appointment.	o	o	o

Please answer the following two questions just to show that you're paying attention to the survey.

Please answer “Slightly unlikely” to this question.

Very unlikely
Unlikely
Slightly unlikely
Slightly likely

Likely
Very likely

What color is the sky? Please answer this question incorrectly, on purpose, by choosing "Red" instead of "Blue."

Blue
Green
Red
Yellow

Please read the following text passage about the J&J/Janssen COVID-19 vaccine. After you read the passage, you will be asked some questions. You may take as much time as you would like to read the passage, and you may look back at the passage as you answer the questions.

***

Recommendation to Pause Use of Johnson & Johnson's Janssen COVID-19 Vaccine

What you need to know:

The use of Johnson & Johnson's Janssen (J&J/Janssen) COVID-19 Vaccine is paused for now. This is because the safety systems that make sure vaccines are safe received a small number of reports of people who got this vaccine experiencing a rare and severe type of blood clot with low platelets. Seek medical care right away if you develop any of the symptoms below. If you have any questions at all, call your doctor, nurse, or clinic.

J&J/Janssen Vaccine Pause Questions and Answers

What if I got the J&J/Janssen COVID-19 Vaccine?

If you got this vaccine more than three weeks ago, your risk of developing a blood clot with low platelets is very low.

If you got this vaccine within the last three weeks, your risk of developing a blood clot with low platelets is also very low. However, you should be on the lookout for possible symptoms of a blood clot with low platelets:

- severe headache
- backache

- blurred vision
- fainting
- seizures
- severe pain in your abdomen or stomach
- severe pain in your chest
- leg swelling
- shortness of breath
- tiny red spots on the skin (petechiae)
- new or easy bruising or bleeding

Should I cancel my vaccination appointment?

If you have an appointment to get the J&J/Janssen COVID-19 Vaccine, please work with your vaccine provider to reschedule your appointment to receive another authorized and recommended COVID-19 vaccine. There are two other COVID-19 vaccines authorized and recommended for use in the United States: Pfizer-BioNTech and Moderna.

What does a pause mean?

On April 13, 2021, CDC and the US Food and Drug Administration (FDA) recommended a pause in the use of J&J/Janssen COVID-19 Vaccine. Although the J&J/Janssen vaccine is still authorized for use, CDC and FDA recommend this vaccine not be given to anyone at this time while this safety signal and its possible implications are investigated. The pause will give scientists a chance to review the data and decide if recommendations on this specific vaccine need to change. CDC and FDA will share more information on this situation as soon as possible.

What do we know?

Scientists and doctors look constantly and carefully at all reported vaccine side effects.

From their monitoring, they saw a small number of reports of people who got the J&J/Janssen COVID-19 Vaccine developing a rare and severe type of blood clot. This type of blood clot is found in the blood vessels that drain blood from the brain and is combined with low platelets. Platelets help blood clot and stop bleeding.

All of these reports were in women between the ages of 18 and 48, and the problems were found up to 2 weeks after vaccination.

There had been more than 7.5 million doses of the J&J/Janssen COVID-19 Vaccine administered as of the time of the pause in the United States.

What are we still learning?

We do not know enough yet to say if the vaccine is related to or caused this health issue. To be extra careful, CDC and FDA recommend that the vaccine not be given until we learn more.

Why did CDC and FDA recommend a pause?

CDC and FDA recommended this pause to give the agencies time to communicate with and prepare the healthcare system to recognize and treat patients appropriately, as well as gather more information about this situation. Communication with healthcare providers is also re-emphasizing the importance of reporting and how to report severe events in people who have received this vaccine. This pause also will allow CDC's independent advisory committee, the Advisory Committee on Immunization Practices, to meet, review these reports, and assess their potential significance.

Report any adverse events after vaccination to v-safe and the Vaccine Adverse Event Reporting System.

What do I need to know about the possible safety issue?

COVID-19 vaccine safety is a top priority for the federal government, and all reports of health problems following COVID-19 vaccination are taken very seriously.

We know the safety systems in place are working. This potential safety issue was caught early, and this pause reflects the federal government's commitment to transparency as CDC and FDA review these data. COVID-19 vaccines have undergone and will continue to undergo the most intensive safety monitoring in U.S. history.

If you or your patients experience any adverse events after vaccination, report them to v-safe and the Vaccine Adverse Event Reporting System.

Has this issue been seen with the other COVID-19 vaccines?

No. As of April 13, 2021, no reports of blood clots with low platelets have been reported among the more than 180 million doses of the Pfizer-BioNTech or Moderna vaccines administered so far.

***

Thinking about the information you just read, how confident are you in the safety of COVID-19 vaccines in general?

Not confident at all
Slightly confident
Somewhat confident
Fairly confident
Completely confident

Thinking about the information you just read, how confident are you in the safety of the J&J/Janssen COVID-19 vaccine in particular?

Not confident at all
Slightly confident
Somewhat confident
Fairly confident
Completely confident

Thinking about the information you just read, how likely would you be to get either the Moderna or Pfizer/BioNTech COVID-19 vaccine?

I definitely would NOT get either the Moderna or Pfizer/BioNTech vaccine.
I probably would NOT get either the Moderna or Pfizer/BioNTech vaccine.
I probably would get either the Moderna or Pfizer/BioNTech vaccine.
I definitely would get either the Moderna or Pfizer/BioNTech vaccine.
I am completely undecided about whether I would get either the Moderna or Pfizer/BioNTech vaccine.
I have already gotten either the Moderna or Pfizer/BioNTech vaccine.
I have already gotten a different COVID-19 vaccine.

Please answer the same three questions as if we had asked you BEFORE the pause of the J&J/Janssen vaccine was announced.

Now imagine that we had asked you these three questions BEFORE the pause of the J&J/Janssen vaccine was announced. How would you have answered the first question, which is how confident are you in the safety of COVID-19 vaccines in general?

Not confident at all
Slightly confident
Somewhat confident
Fairly confident
Completely confident

Now imagine that we had asked you these three questions BEFORE the pause of the J&J/Janssen vaccine was announced. How would you have answered the second question, which is how confident are you in the safety of the J&J/Janssen COVID-19 vaccine in particular?

Not confident at all
Slightly confident
Somewhat confident
Fairly confident
Completely confident

Now imagine that we had asked you these three questions BEFORE the pause of the J&J/Janssen vaccine was announced. How would you have answered the third question, which is how likely would you be to get either the Moderna or Pfizer/BioNTech COVID-19 vaccine if available to you?

I definitely would NOT get either the Moderna or Pfizer/BioNTech vaccine.
I probably would NOT get either the Moderna or Pfizer/BioNTech vaccine.
I probably would get either the Moderna or Pfizer/BioNTech vaccine.
I definitely would get either the Moderna or Pfizer/BioNTech vaccine.
I am completely undecided about whether I would get either the Moderna or Pfizer/BioNTech vaccine.
I have already gotten either the Moderna or Pfizer/BioNTech vaccine.
I have already gotten a different COVID-19 vaccine.

Using a six-point scale, please indicate how strongly you agree with each of the following statements about the passage.

I think that the information in the passage is accurate and should be trusted.

Strongly disagree
Disagree
Slightly disagree
Slightly agree

Agree
Strongly agree

I think that the information in the passage is based on high-quality evidence.

Strongly disagree
Disagree
Slightly disagree
Slightly agree
Agree
Strongly agree

I think that the writing in the passage is clear and easy to read.

Strongly disagree
Disagree
Slightly disagree
Slightly agree
Agree
Strongly agree

I think that I understand the information in the passage.

Strongly disagree
Disagree
Slightly disagree
Slightly agree
Agree
Strongly agree

I think that I had to put a lot of effort into understanding the information in the passage.

Strongly disagree
Disagree
Slightly disagree
Slightly agree
Agree
Strongly agree

I think that other people would want to read the passage.

Strongly disagree
Disagree
Slightly disagree
Slightly agree
Agree
Strongly agree

I think that I would share the information in the passage with friends on social media.

Strongly disagree
Disagree
Slightly disagree
Slightly agree
Agree
Strongly agree

Please answer the following questions with your best guess.

As you read in the passage, more than 7.5 million people have received the J&J/Janssen vaccine in the U.S. *If you had to guess*, about how many of them have developed a rare and severe type of blood clot after being vaccinated?

1 person
10 people
100 people
1,000 people
10,000 people
100,000 people
1,000,000 or more people

As you read in the passage, more than 7.5 million people have received the J&J/Janssen vaccine in the U.S. *If you had to guess*, about how many of them have died from a rare and severe type of blood clot after being vaccinated?

0 people
1 person
10 people
100 people
1,000 people

10,000 people
100,000 people
1,000,000 or more people

If you had to guess, how much longer do you think the pause in use of the J&J/Janssen COVID-19 vaccine will last?

1 day
2-6 days
1-2 weeks
2-4 weeks
1-2 months
More than 2 months (but not permanent)
I think the pause will be permanent.

Thank you for reading the text passage about the J&J/Janssen COVID-19 vaccine and answering our questions.

Now please answer a second set of questions based only on the information in the same passage that you just read. As before, you may take as much time as you would like to reread the passage, and you may look back at the passage as you answer the questions.

Is it currently legal to give the J&J/Janssen vaccine in the U.S.?

No, because the FDA has removed its emergency authorization.
No, because its use has been paused by the FDA and CDC.
Yes, because the pause in use is a recommendation, not a requirement.
The passage does not say.

Why are the CDC and FDA recommending a pause in use of the J&J/Janssen vaccine?

After being approved for emergency use in the U.S., the vaccine was found to be ineffective at preventing COVID-19 infections, hospitalizations, and deaths.
Problems were recently identified with how the original clinical trial was run.
J&J has had trouble making the vaccine, and almost no doses are available now.
A possible safety issue has been identified with the vaccine, and more time is needed to study the issue.

Does the J&J/Janssen COVID-19 vaccine cause blood clots?

No
Yes
More information is needed to know for sure.

A rare and severe type of blood clot has been reported in ________ who received the J&J/Janssen vaccine.

women
men
both men and women
people over the age of 50

On April 1st John got his first shot of the Pfizer/BioNTech vaccine. The day after getting vaccinated, he felt tired and had a mild headache. His second shot is scheduled for this week. Should John cancel the appointment because of safety concerns?

No, because a rare and severe type of blood clot has only been reported in people who received the J&J/Janssen vaccine, not people who received the Moderna or Pfizer/BioNTech vaccines.
No, because blood clots are only caused by the first dose of Pfizer/BioNTech vaccine, not the second.
Yes, because he had a headache after the first dose.
Yes, because possible safety issues with the J&J/Janssen vaccine might apply to all COVID-19 vaccines.

Elizabeth got the J&J/Janssen COVID-19 vaccine six weeks ago. Since then she has not had any side effects from the vaccine or symptoms of COVID-19. In light of the pause, she should ________.

get the Moderna vaccine
get the Pfizer/BioNTech vaccine
ask her doctor for advice about getting a different vaccine

Linda gets the J&J/Janssen vaccine on Monday. On Friday she develops a severe headache. What should she do first?

Seek urgent medical care for a possible blood clot.
Monitor herself for 24 hours and then seek medical care if the headache has not improved.
Do nothing, because headaches are a normal side effect of COVID-19 vaccines.
Report the symptom through v-safe.

Jessie's appointment to get the J&J/Janssen vaccine has been canceled because of the pause. What should they do now?

Work with their vaccine provider to reschedule the appointment and get a different COVID-19 vaccine.
Wait to get vaccinated until the J&J/Janssen vaccine is available again.
Show up for the canceled appointment and ask to get a different vaccine.
Either the first or the second answer is correct.

Please answer the following questions about your background.

How old are you?

18-29
30-39
40-49
50-64
65 or older

Which best describes your gender? Please choose as many options as apply.

Female
Male
Non-binary
Transgender
Another option not listed here (please specify): ______________________________

Are you Hispanic or Latino/Latina/Latinx?

Not Hispanic or Latino/Latina/Latinx
Hispanic or Latino/Latina/Latinx

Which best describes your race? Please choose as many options as apply.

American Indian or Alaska Native
Asian
Black or African American
Native Hawaiian or Other Pacific Islander
White

Another option not listed here (please specify): ____

What is your highest level of formal education?

Some high school or less
High school diploma or equivalent
Some college or associate's degree
Bachelor's degree
Graduate or professional degree

Generally speaking, which of the following best describes your political affiliation?

Strong Republican
Leaning Republican
Leaning Democratic
Strong Democratic
Independent
Another option not listed here (please specify): ____

Which candidate did you vote for in the 2020 U.S. presidential election?

Joe Biden (Democratic)
Donald Trump (Republican)
Jo Jorgensen (Libertarian)
Howie Hawkins (Green)
I voted for someone else not listed here.
I did not vote.

Thinking about your general approach to issues, do you consider yourself to be ________?

Very conservative
Somewhat conservative
Moderate
Somewhat liberal
Very liberal
Not sure

In which state do you live?

▼ Alabama ... District of Columbia

How would describe the area where you live?

Rural area
Suburban area
Urban area

Did you use Google or any other outside sources to answer the questions? Please answer honestly. Your payment does NOT depend on your response to this question.

No
Yes

How carefully did you complete this survey? Please answer honestly. Your payment does NOT depend on your response to this question.

Not at all carefully
Slightly carefully
Moderately carefully
Carefully
Very carefully

You might find this information to be useful.

CDC and FDA have recommended a pause in the use of the Janssen (Johnson & Johnson) COVID-19 vaccine in the United States out of an abundance of caution, effective Tuesday, April 13.

Scientists and doctors look constantly and carefully at all reported vaccine side effects.

About 7 million Janssen COVID-19 vaccine doses administered in the United States to date, six (6) cases of a rare and severe type of blood clot called "cerebral venous sinus thrombosis" (CVST) were seen in combination with low levels of blood platelets (thrombocytopenia). This type of blood clot is found in the blood vessels that drain blood from the brain and is combined with low platelets. Platelets help blood clot and stop bleeding.

All six (6) cases of the "rare and severe" blood clots occurred in women between the ages of 18 and 48, and the problems were found up to 2 weeks after vaccination.

When these specific types of blood clots are observed following J&J COVID-19 vaccination, treatment is different from the treatment that might typically be administered for blood clots. The purpose of this Health Alert is, in part, to ensure that the healthcare provider community is aware of the potential for these adverse events and can provide proper management due to the unique treatment required with this type of blood clot.

The U.S. Food and Drug Administration (FDA) issued Emergency Use Authorization (EUA) to the single-dose Johnson & Johnson's Janssen COVID-19 vaccine, to prevent COVID-19 in individuals 18 years of age and older on Feb 27th 2021. Of all the Janssen COVID-19 vaccine doses administered in the United States to date, the most common side effects with are usually mild or moderate and get better within 1 or 2 days after vaccination.

Thank you for taking the survey.
