# Supplementary material for: Response of Unvaccinated US Adults to Official Information About the Pause in Use of the Johnson & Johnson–Janssen COVID-19 Vaccine: Cross-Sectional Survey Study
Source: J Med Internet Res. 2024 Apr 1;26:e41559. doi: 10.2196/41559 (PMC11019423; doi:10.2196/41559)
Supplement: Multimedia Appendix 4 [file jmir_v26i1e41559_app4.pdf]

Multimedia Appendix 4: Supplemental tables about survey questions and ordinal logistic regression analysis

Table A4.1. Full responses to comprehension questions.

Question, Answer Choices[a]	Cohort A (*N* = 271) No. (%)	Cohort B (*N* = 286) No. (%)
Why are the CDC and FDA recommending a pause in use of the J&J-Janssen vaccine?		
(1) A possible safety issue has been identified with the vaccine, and more time is needed to study the issue.*	259 (95.6)	280 (97.9)
(2) After being approved for emergency use in the U.S., the vaccine was found to be ineffective at preventing COVID-19 infections, hospitalizations, and deaths.	4 (1.5)	2 (0.70)
(3) Problems were recently identified with how the original clinical trial was run.	4 (1.5)	4 (1.4)
(4) J&J has had trouble making the vaccine, and almost no doses are available now.	4 (1.5)	0 (0)
Does the J&J-Janssen COVID-19 vaccine cause blood clots?		
(1) More information is needed to know for sure.*	196 (72.3)	215 (75.2)
(2) No	3 (1.1)	4 (1.4)
(3) Yes	72 (26.6)	67 (23.4)
A rare and severe type of blood clot has been reported in _______ who received the J&J-Janssen vaccine.		
(1) women*	234 (86.3)	248 (86.7)
(2) men	1 (0.4)	0 (0)
(3) both men and women	28 (10.3)	36 (12.6)
(4) people over the age of 50	8 (3.0)	2 (0.7)
On April 1st John got his first shot of the Pfizer-BioNTech vaccine. The day after getting vaccinated, he felt tired and had a mild headache. His second shot is scheduled for this week. Should John cancel the appointment because of safety concerns?		
(1) No, because a rare and severe type of blood clot has only been reported in people who received the J&J-Janssen vaccine, not people who received the Moderna or Pfizer-BioNTech vaccines.*	214 (79.0)	250 (87.0)
(2) No, because blood clots are only caused by the first dose of Pfizer-BioNTech vaccine, not the second.	5 (1.8)	3 (1.0)
(3) Yes, because he had a headache after the first dose.	18 (6.6)	14 (4.9)
(4) Yes, because possible safety issues with the J&J-Janssen vaccine might apply to all COVID-19 vaccines.	34 (12.5)	19 (6.6)

Elizabeth got the J&J-Janssen COVID-19 vaccine six weeks ago. Since then she has not had any side effects from the vaccine or symptoms of COVID-19. In light of the pause, she should ________.		
(1) do nothing different*	164 (60.5)	189 (66.1)
(2) get the Moderna vaccine	1 (0.4)	1 (0.3)
(3) get the Pfizer-BioNTech vaccine	8 (3.0)	8 (2.8)
(4) ask her doctor for advice about getting a different vaccine	98 (36.2)	88 (30.8)
Linda gets the J&J-Janssen vaccine on Monday. On Friday she develops a severe headache. What should she do first?		
(1) Seek urgent medical care for a possible blood clot.*	180 (66.4)	121 (42.3)
(2) Monitor herself for 24 hours and then seek medical care if the headache has not improved.	62 (22.9)	87 (30.4)
(3) Do nothing, because headaches are a normal side effect of COVID-19 vaccines.	6 (2.2)	4 (1.4)
(4) Report the symptom through v-safe.	23 (8.4)	74 (25.9)
Jessie's appointment to get the J&J-Janssen vaccine has been canceled because of the pause. What should they do now?[b]		
(1) Work with their vaccine provider to reschedule the appointment and get a different COVID-19 vaccine.*	192 (70.8)	229 (80.1)
(2) Wait to get vaccinated until the J&J-Janssen vaccine is available again.	10 (3.7)	6 (2.1)
(3) Show up for the canceled appointment and ask to get a different vaccine.	9 (3.3)	4 (1.4)
(4) Either the first or the second answer is correct.	60 (22.1)	47 (16.4)

[a]Unless otherwise indicated, answer choices were presented to the participants in a random order. For clarity the correct answer is always listed first in the table and marked with an asterisk.
[b]Order of answer choices was not randomized.

Table A4.2. Summary of ordinal logistic regression analysis.

Feature	Odds Ratio	95% CI	*P*
**Cohort A**			
Age			
≥50	0.94	0.64-1.39	.77
Gender			
Female	1.18	0.92-1.53	.20
Ethnicity			
Hispanic or Latinx	0.83	0.59-1.17	.30
Race			
Asian	1.13	0.72-1.77	.61
Black or African American	0.78	0.56-1.08	.13
Other	1.16	0.77-1.75	.48
Educational attainment			
High school diploma or less	0.66	0.48-0.89	.007
Political partisanship			
Republican	1.25	0.93-1.69	.14
Geography			
Rural area	1.12	0.77-1.63	.54
Intention to receive vaccine			
Definitely will not	0.61	0.45-0.82	.001
**Cohort B**			
Age			
≥50	1.26	0.83-1.91	.28
Gender			
Female	1.33	1.03-1.71	.029
Ethnicity			
Hispanic or Latinx	1.09	0.71-1.65	.70
Race			
Asian	0.85	0.62-1.15	.28
Black or African American	0.46	0.31-0.68	<.001
Other	0.85	0.53-1.35	.48
Educational attainment			
High school diploma or less	0.79	0.57-1.09	.15
Political partisanship			
Republican	0.85	0.59-1.23	.56
Geography			
Rural area	0.89	0.60-1.32	.39

Intention to receive vaccine			
Definitely will not	0.48	0.31-0.74	.001
